# Spotlight on CYP4B1

**DOI:** 10.3390/ijms24032038

**Published:** 2023-01-20

**Authors:** Annika Röder, Saskia Hüsken, Michael C. Hutter, Allan E. Rettie, Helmut Hanenberg, Constanze Wiek, Marco Girhard

**Affiliations:** 1 Institute of Biochemistry, Heinrich Heine University, 40225 Düsseldorf, Germany; 2 Department of Otorhinolaryngology and Head/Neck Surgery, Heinrich Heine University, 40225 Düsseldorf, Germany; 3 Center for Bioinformatics, Saarland University, 66123 Saarbrücken, Germany; 4 Department of Medicinal Chemistry, School of Pharmacy, University of Washington, Seattle, DC 98105, USA; 5 Department of Pediatrics III, University Children’s Hospital Essen, University Duisburg-Essen, 45147 Essen, Germany

**Keywords:** cytochrome P450, CYP4B1, structure, bioactivation, xenobiotics, drug metabolism, endobiotic metabolism, cancer, suicide gene

## Abstract

The mammalian cytochrome P450 monooxygenase CYP4B1 can bioactivate a wide range of xenobiotics, such as its defining/hallmark substrate 4-ipomeanol leading to tissue-specific toxicities. Similar to other members of the CYP4 family, CYP4B1 has the ability to hydroxylate fatty acids and fatty alcohols. Structural insights into the enigmatic role of CYP4B1 with functions in both, xenobiotic and endobiotic metabolism, as well as its unusual heme-binding characteristics are now possible by the recently solved crystal structures of native rabbit CYP4B1 and the p.E310A variant. Importantly, CYP4B1 does not play a major role in hepatic P450-catalyzed phase I drug metabolism due to its predominant extra-hepatic expression, mainly in the lung. In addition, no catalytic activity of human CYP4B1 has been observed owing to a unique substitution of an evolutionary strongly conserved proline 427 to serine. Nevertheless, association of CYP4B1 expression patterns with various cancers and potential roles in cancer development have been reported for the human enzyme. This review will summarize the current status of CYP4B1 research with a spotlight on its roles in the metabolism of endogenous and exogenous compounds, structural properties, and cancer association, as well as its potential application in suicide gene approaches for targeted cancer therapy.

## 1. Cytochrome P450 Monooxygenases and CYP4B1: A Short Survey

Cytochrome P450 monooxygenases (P450s) are heme-thiolate proteins capable of catalyzing a large variety of chemically challenging reactions upon the reductive cleavage of molecular oxygen. The most common reaction is the hydroxylation of non-activated C-H bonds in organic molecules according to the general reaction R-H + O_2_ + NAD(P)H + H^+^ → R-OH + NAD(P)^+^ +H_2_O [1]. Other common P450-catalyzed reactions include epoxidation of C=C double bonds, N- and S-oxidations, and N-, O-, or S-dealkylations [2]. In addition, some uncommon reaction types such as aromatic/phenolic coupling, bond cleavage (e.g., C-C-lyase reaction) or migration, ring opening and closure, and the rearrangement of either oxidized products or the involvement of enzyme intermediates can also be observed, leading to a higher level of chemical space expansion for natural products [3,4].

P450s are often able to act in a stereo- and regiospecific manner and when combined with a tremendous number of known substrates, these features make them powerful biocatalysts for specific biotransformation reactions in drug development and improvement, as well as biosynthetic applications [5,6]. Since the first discovery of a P450 about 70 years ago [7], the number of P450 sequences has been growing almost exponentially. Today P450s constitute one of the largest enzyme superfamilies with more than 1,000,000 known sequences spanning all domains of life (Bacteria, Archea, Eukarya) [8] and even viruses [9].

In order to unequivocally classify the growing number of P450 sequences, a systematic nomenclature based on sequence identity has been used since 1993 [10]. Therein, the term CYP (from Cytochrome P450) is followed by a number indicating the family (sequence identity of > 40%), followed by a letter for the subfamily (sequence identity > 55%). Within a subfamily, the enzymes are consecutively numbered in the order of their discovery.

This review puts the spotlight on one particular P450, namely, mammalian CYP4B1. This predominantly extrahepatic P450 was first extracted from rabbit lung tissue in the 1970s, where it was identified along with CYP2B4 as the dominant microsomal P450, accounting for at least 35% of the total P450s [11,12,13]. As one of 58 human P450 isoenzymes (of which 57 are encoded in the human genome; the 58th—CYP4F3B—is alternatively spliced), CYP4B1 belongs to the CYP4 family, which also includes the CYP4A, 4F, 4V, 4X, and 4Z subfamilies [14]. Enzymes in the CYP4 family in mammals are generally involved either in endobiotic metabolism acting on fatty acids and signaling molecules including eicosanoids, or the modification of xenobiotics and therapeutic drugs. Consequently, CYP4 family members have been implicated in various biological functions, including among others inflammation, skin barrier, eye function, cardiovascular health, and cancer [15,16,17]. Furthermore, the CYP4 family is one of the oldest P450 families whose members have usually been highly conserved during evolution [18].

**Figure 1:**
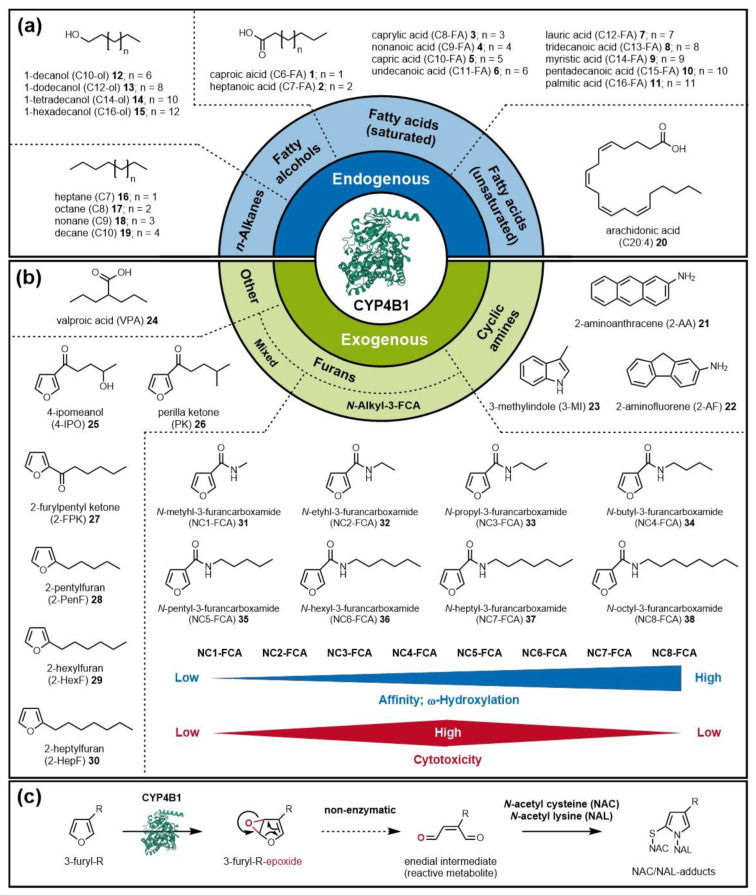
Overview of CYP4B1 substrates. (**a**) Endogenous substrates that are hydroxylated by CYP4B1 include fatty acids **1**–**11, 20**, fatty alcohols **12**–**15**, and *n*-alkanes **16**–**19**. (**b**) Exogenous substrates of CYP4B1 can be grouped into cyclic amines **21**–**23**, VPA **24**, and furyl-containing compounds **25**–**38**. (**c**) Schematic representation of the bioactivation pathway of 3-furyl-containing compounds **25, 26, 31**–**38**. CYP4B1 catalyzes the epoxidation of the furan ring, followed by non-enzymatic rearrangement to an enedial intermediate. In vivo, these reactive enedial intermediates bind to macromolecules (e.g., proteins) causing cytotoxicity; in vitro, they can be trapped by reactions with nitrogen and sulfur nucleophiles (e.g., NAC/NAL) that generate stable S-substituted pyrroles.

CYP4B1 is special in the CYP4 family (along with CYP4A11) because, unlike the other members, it acts at the interface of endo- and xenobiotic metabolism. Thus, CYP4B1 can hydroxylate common endobiotic substrates such as fatty acids (Figure 1a), as well as activate xenobiotics such as 4-ipomeanol (4-IPO) **25**, perilla ketone (PK) **26** or valproic acid (VPA) **24** (Figure 1b) [19,20,21,22,23].

Strikingly, this situation is completely different in humans. In contrast to other species studied to date, no catalytic activity has been demonstrated for the native human enzyme (hCYP4B1) so far. It is suspected that at least the exchange of one amino acid—a highly conserved proline residue at position 427 to serine—leads to the apparent inactivity of hCYP4B1 [24]. Thus, the physiological function of CYP4B1 in humans is still unclear and consequently it is classified as an “orphan P450”. On the opposite side, apart from its unknown physiological function, hCYP4B1 is thought to be involved in several types of cancer due to altered gene expression levels in tumor tissues compared to normal tissue [25,26,27].

Since rabbit CYP4B1 (rCYP4B1), unlike hCYP4B1, is known to activate exogenous protoxins such as 4-IPO **25**, resulting in highly toxic compounds (Figure 1c), it has been considered a promising candidate for the clinical application of tumor-killing suicide gene systems [28,29].

Together with other topics such as tissue distribution and gene regulation (last reviewed by Lim et al. [30]), this review will also cover the current status of the biochemistry and structure of CYP4B1, which was last reviewed in 2006 [31].

## 2. Structural Properties of CYP4B1

Recently, three X-ray crystallographic structures of rCYP4B1 were reported [32,33]. These comprise the native enzyme (5T6Q.pdb and 6C94.pdb) and the p.E310A mutant (6C93.pdb). While the heme prosthetic group (protoporphyrin IX) of P450s is generally coordinated by a cysteine thiolate which dictates the spectral behavior of P450s, and is additionally stabilized in the apoprotein by electrostatic forces, hydrogen bonds, and ligand coordination [34,35], some CYP4 members (including CYP4B1) differ in that they have a covalent ester link between the heme 5-methyl and a glutamic acid residue, e.g., members of the subfamilies CYP4A, CYP4B, and CYP4F in human, rabbit, and rat were found to bind their heme group covalently [36,37,38]. In rCYP4B1, the heme moiety is covalently linked to the side chain of E310, which is part of the I-helix [32]. This ester linkage is formed auto-catalytically and is in turn cleavable by acids and bases [36,39].

Together with the additional restraint exerted by the heme 7-propionate, the resulting shape of the binding pocket directs endogenous substrates in a preferential orientation for ω-hydroxylation, particularly in the case of shorter hydrocarbon chains. However, hydroxylation is also observed in positions ω-1 and ω-2 if the terminal part of the carbon chain is able to fold within the binding pocket [23,39]. This was previously also observed in computational studies, where fatty acids with chain lengths C8 to C15 **3**–**10** were docked into homology models of rCYP4B1 and hCYP4B1 [23]. With increasing chain length, possibilities for hydroxylation up to position ω-10 were found because the U-shaped folding of the hydrocarbon chains brings the α-, β-, γ-, and δ-positions close to the iron of the heme group. Furthermore, computed activation barriers for hydrogen abstraction at carbon atoms of lauric acid (C12-FA) **7** and 1-dodecanol (C12-ol) **13** indicate that there is no energetically preferred position along the carbon chain, and therefore the hydroxylation reaction is kinetically controlled [23].

Besides the ω-hydroxylation of alkane chains, the epoxidation of aromatic moieties was observed, e.g., of 4-IPO **25** and PK **26**. Corresponding orientations of the furan ring in the active center were also seen in docking simulations [23].

CYP4B1 is wide-spread among organisms, and the number of sequences that have been annotated in UniProt is greater than 600 (https://www.uniprot.org/, accessed on 16 June 2022). Although the amino acids along the I-helix are strongly conserved throughout these species, E310 is frequently replaced by either alanine or glycine. This is mostly seen in tropical birds but also in some mammals, such as the domestic goat, the African elephant, and the olive baboon. In the p.E310A mutant of rCYP4B1, a strong shift in regioselectivity (decreased ω-hydroxylation, increased ω-1 hydroxylation) was observed, while the overall rate of metabolism was retained. The shift in regioselectivity was therefore attributed to the reduced rigidity of the heme vicinity [33].

**Figure 2:**
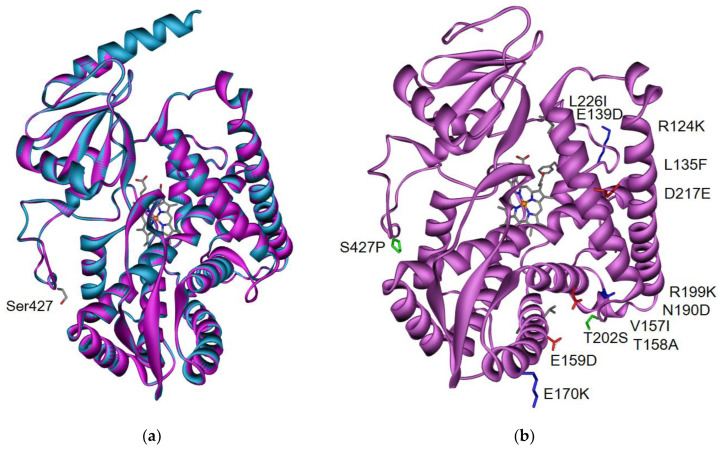
(**a**) Sequence and structure comparison between native rCYP4B1 (cyan) and the homology model of native hCYP4B1 (mauve): Location of S427 of hCYP4B1 is highlighted (**b**) Homology model of engineered h-P+12: Nonidentical amino acids compared to native hCYP4B1 are depicted (green: hydrophilic, grey: hydrophobic, red: negatively charged, blue: positively charged). Please note that the engineered h-P+12 does not contain parts of the original N-terminus (first α-helix).

Sequence conservation between rCYP4B1 and hCYP4B1 is high (86.2% identity), and most importantly, the amino acids in the active center are identical (Figure S1). Therefore, the crystallographic structures of rCYB4B1 are suitable templates for homology modeling of hCYP4B1 (Figure 2a) [23]. Nevertheless, CYP4B1 sequences of apes and other mammals (e.g., cat, dog, horse, donkey, but not mouse) are more closely related. A comparison of the homology models for hCYP4B1 generated using Swiss Model (https://swissmodel.expasy.org, accessed on 25 January 2021) and AlphaFold (https://alphafold.ebi.ac.uk/, accessed on 21 December 2021) showed a root-mean-square deviation of atomic positions (RMSD) for the backbone atoms of 0.45 Å. For comparison, the corresponding RMSD between the two crystallographic structures of native rCYP4B1 (6C94.pdb and 5T6Q.pdb) is 0.33 Å.

Striking is the substitution of proline 427 by serine in hCYP4B1 that is unique among all known CYP4B1 sequences (Figure S2). This residue is located on the surface of the protein and therefore is far from the heme group (>15 Å) (Figure 2a). Since proline is known for its rigidity, thus influencing the flexibility of the protein backbone, it was suggested that it stabilizes this so-called ‘meander region’ in contrast to S427, which is at the start of a short α-helix [40]. Reestablishing P427 together with 12 additional mutations (R124K/E130D/L135F/V154I/T158A/E159D/E170K/N190D/R199K/T202S/D217E/L226I) is thought to improve the structural stability due to enhanced hydrogen-bonding or salt bridges, resulting in a stable enzyme (h-P+12; Figure 2b and Figure S3) that exhibits 4-IPO **25** metabolizing activity similar to rCYP4B1 [40].

## 3. Metabolic Activities of CYP4B1

### 3.1. Endogenous CYP4B1 Substrates

As one of the oldest P450 families, the CYP4 family (Figure S3) evolved about 1.25 billion years ago. CYP4s are evolutionarily related to cholesterol-metabolizing enzymes that appeared at the same time, and hence they were suggested to have been involved together in maintaining membrane integrity in early eukaryotes [18,41]. In the literature, the enzymes of the mammalian CYP4 family are generally referred to as fatty acid hydroxylases; thus, by definition, their function is to facilitate the removal of excess free fatty acids by the addition of oxygen to a terminal C-H to prevent lipotoxicity [15,16,42]. Generally, the resulting ω-alcohols are further oxidized to yield dicarboxylic acids, but alternatively they can also be converted into glucuronides or be esterified to glycerolipids [43]. CYP4 enzymes also participate in the metabolism of branched-chain fatty acids and eicosanoids [15,16,44]. They generally have a high preference for hydroxylation of the carbon at the terminal end (ω-position) of a poly-hydrocarbon chain [45]. Since the terminal C-H methyl bond is stronger than that of other positions of fatty acids and thus energetically unfavored [23], most other P450s catalyze the energetically favored ω-1 or more remote ω-n hydroxylation(s) [46,47,48,49]. The unfavorable selectivity for terminal hydroxylation requires that CYP4 proteins actively influence it. Two research groups independently reported an ester link between a glutamic acid of the protein backbone and the heme prosthetic group distinguishes most CYP4 proteins from other P450s and probably leads to this preference [36,37,50]. As mentioned above, E310 in rCYP4B1 plays a crucial role in its preference for ω-hydroxylation, as verified by mutation experiments, wherein, for example, the p.E310A variant had the same overall turnover rate of octane (C8) **17** in comparison to the native enzyme, but a fourfold reduced regioselectivity for ω-hydroxylation [39].

P450-catalyzed ω-hydroxylation is also associated with the formation of bioactive metabolites from arachidonic acid (C20:4) **20** and likewise the degradation of signaling molecules such as the tissue hormones leukotrienes and prostanoids. One study reported that 20-hydroxyeicosatetraenoic acid (20-HETE) can be formed by rCYP4B1, but this could not be confirmed by other research groups [16,51]. Nevertheless, the preference for catalyzing ω- over ω-1-hydroxylation may have physiological consequences in this case: The ω-hydroxylation product of C20:4 **20** results in the vasoconstrictor 20-HETE, while ω-1 hydroxylation results in the vasodilator 19-HETE [52,53,54,55,56]. Under certain circumstances, CYP4B1 has also been described to be involved in the cornea in the conversion of C20:4 **20** to 12-hydroxy-5,8,14-eicosatrienoic acid (12-HETrE) by ω-9-hydroxylation [57].

With regard to fatty acid hydroxylation, Muerhoff et al. were the first to show that at least two C12-FA **7** hydroxylases exist in rabbit lung microsomes [58]. For this purpose, they used so-called suicide substrates such as 1-aminobenzotriazole and allylisopropylacetamide that inhibit microsomal P450-dependent activities. They were able to demonstrate that rCYP4B1, which was one of the two mentioned rabbit lung microsomal P450s, hydroxylates C12-FA **7**, which was later confirmed by Guan et al. using the recombinant protein [58,59]. The latter group also investigated desaturation reactions of C12-FA **7** catalyzed by different rabbit P450s and observed that CYP2E1 prefers ω-1-hydroxylation, while CYP4A5/A6 as well as rCYP4B1 prefer ω-hydroxylation, and that in addition an olefinic product, 11-ene-lauric acid was formed mainly in the reaction with rCYP4B1 [59].

To investigate whether the regioselectivity of CYP4B1 is dependent on the fatty acid chain length, Fisher et al. tested rCYP4B1 recombinantly expressed in insect cells with short- to medium-chain fatty acids (C7-FA to C10-FA) **2–5** as well as *n*-alkanes of the same chain lengths (C7 to C10) **16–19** [60]. In the case of fatty acids, C7-FA **2** was a poor substrate for rCYP4B1, whereas C8-FA to C10-FA **3–5** were converted at rates around 11 min^−1^, and ω-hydroxylation was preferred in all three cases. However, it was observed that regioselectivity decreased with increasing chain length; C8-FA **3** had an ω/ω-1 ratio of 7.4, while for C10-FA **5**, an ω/ω-1 ratio of 1.1 was observed. The *n*-alkanes C7 to C10 **16–19** were rapidly converted in a regioselective manner (C10 **19**: 11 min^−1^ to C7 **16**: 33 min^−1^). Again, the preference for ω-hydroxylation increased with decreasing chain length; for C7 **16**, the highest selectivity (ω/ω-1 ratio of 23) was observed, which decreased to 1.6 in the case of C10 **19** [60]. Based on these results, it was hypothesized that C8-FA **3** and C7 **16** have the optimal chain length for regioselective ω-hydroxylation by rCYP4B1 and that hydrocarbons are generally more rapidly converted than fatty acids. This was later confirmed by similar results from Hsu et al., who reconstituted the activity of rCYP4B1 that was recombinantly expressed and purified from *E. coli* [32]. Using the resolved crystal structure of rCYP4B1 complexed with C8 **17**, the authors also showed that the active site cavity is almost filled by this compound. Therefore, they suggested that longer chain lengths caused the cavity size to expand, which is why the regioselectivity might decrease [32]. Comparisons with other ω-hydroxylases of the CYP4 family show that they differ in their preferred chain lengths to act in a regiospecific manner: For CYP4A1 as one example, C12-FA **7** provides the highest ω-regioselectivity, whereas rCYP4B1 shows only modest regioselectivity for C12-FA **17** (ω/ω-1 = 1.4) [60,61].

More recently, fatty acids as well as fatty alcohols with chain lengths C9 to C16 (**4–11** and **12–15**) were tested with two CYP4B1 isoforms recombinantly expressed in *E. coli*, namely, rCYP4B1 and the reconstituted hCYP4B1 S427P variant (h-S427P) [23,62]. In addition to the previously described ω- and ω-1-hydoxylations on fatty acids, products from the oxidation of other C-H positions were also found. Both rCYP4B1 and h-S427P were able to carry out α-, β-, γ-, and δ-hydroxylations on C10-FA to C14-FA **5–9** with a preference for α- over β- and γ- over δ-positions. After reaction with C12-FA **7** for which the highest conversion was observed, seven reaction products were detected via gas chromatography coupled to mass spectrometry (GC/MS)—a most diverse product pattern that also included ω-, ω-1-, and ω-2-hydroxy fatty acids in addition to the aforementioned new products. Notably, γ- and δ-dodecalactone were also detected (albeit in low amounts) that were probably formed non-enzymatically from the CYP4B1-generated γ- and δ-hydroxy products. Nevertheless, ω-hydroxylation was preferred for most fatty acids tested (C9 to C15) **4–10** for both rCYP4B1 and h-S427P [23].

When both recombinant CYP4B1 isoforms were tested with fatty alcohols C10-ol to C16-ol **12–15**, exclusively ω-, ω-1, and ω-2-hydroxylations were observed. rCYP4B1 showed the highest conversion of C12-ol **13** while in the case of h-S427P, the conversions of C10-ol **12** and C12-ol **13** were comparably high. Notably, the regioselectivity differed in the case of fatty alcohols: rCYP4B1 generally preferred the ω-1 and ω-2 positions, while h-S427P had a higher preference for ω-hydroxylation. Moreover, neither of the C16 compounds (C16-FA **11** and C16-ol **15**) could be converted by either rCYP4B1 or h-S427P [23].

In summary, albeit differences in substrate oxidation rates and regioselectivities were observed, the profiles of converted endogenous substrates are generally similar for rCYP4B1 and h-S427P.

### 3.2. Exogenous CYP4B1 Substrates

CYP4B1 is also known for its ability to activate xenobiotics, which can result in toxic consequences. The anticonvulsant drug VPA **24** is a non-naturally occurring branched carboxylic acid that is used to treat epilepsy and bipolar disorders [63]. High VPA **24** desaturation was reported when testing rabbit lung microsomes that was reduced by more than 80% when antibodies against CYP4B1 were used [21]. The second main rabbit lung microsomal P450 expressed at approximately the same concentration is CYP2B4. When antibodies against CYP2B4 were used in the same experiment, the decrease in product formation was only 15%. This indicated that VPA **24** conversion probably originates from CYP4B1. After the recombinant expression of rCYP4B1 in HepG2 cells and subsequent reaction with VPA **24**, the products 5-hydroxy-, 4-hydroxy-, and the hepatotoxic olefinic metabolite 4-ene-VPA were identified in a ratio of 110:2:1 [21]. This observation confirmed the high preference of rCYP4B1 for ω-hydroxylation over ω-1-hydroxylation. Interestingly, none of the VPA **24** metabolites was detected during testing of CYP4A1 and CYP4A3 that represent other CYP4 family members, indicating a unique property of CYP4B1 in that family [21].

The bioactivation of xenobiotics by CYP4B1 is frequently associated with pneumotoxins, as the enzyme is largely produced in the lungs, more precisely in nonciliated bronchiolar epithelial Clara cells. The most prominent example of a protoxin is 4-IPO **25**. This furan derivate is formed as a defense mechanism in the common sweet potato *Ipomoea batatas* when it is infected with the mold *Fusarium solani* [64]. 4-IPO **25** was first described as a toxin in the early 1970s when cattle showed signs of poisoning after eating sweet potatoes. The animals experienced respiratory distress such as pulmonary edemas that resulted in their subsequent death [65]. In laboratory animal studies, the same symptoms were observed in other mammals such as mice, rats, and dogs. The toxicity effects were predominantly confined to the lungs. Mild liver poisoning was detected only when high doses were administered, and kidney poisoning was detected only in male mice [66,67,68]. It was hypothesized that the covalent binding of alkylated metabolites of 4-IPO **25** to proteins of Clara cells results in pulmonary edema and necrosis [69]. The first link between 4-IPO **25** and rCYP4B1 was made by Slaughter et al. when they observed the inhibition of covalent binding by using CYP4B1 antibodies [70]. Later, the binding of 4-IPO **25** to cellular DNA in human HepG2 cells infected with an rCYP4B1-cDNA-containing vaccinia virus demonstrated that rCYP4B1 can bioactivate 4-IPO **25** to reactive DNA-binding metabolites [71]. That the interaction of these metabolites has a cytotoxic effect was first observed by studies in mouse cell lines [72]. Examination of the tissue distribution of CYP4B1 in rabbit showed that more than 40% of the enzyme was found in the lung with only very small amounts in the liver [21]. Thus, a connection to the almost exclusive pneumotoxicity of 4-IPO **25** in rabbits could be established. With the use of purified rCYP4B1, the bioactivation pathway of 4-IPO **25** was identified using N-acetyl cysteine (NAC) and N-acetyl lysine (NAL) as exogenous nucleophiles to trap the reactive intermediate in vitro [19]. CYP4B1 transfers oxygen to the furan ring of 4-IPO **25**, which then undergoes non-enzymatic rearrangement, generating a highly toxic alkylating enedial metabolite (Figure 1c). In vivo, these events provoke DNA–protein cross-links and DNA strand breaks, resulting in apoptosis. However, this conversion of 4-IPO **25** cannot be catalyzed (<1%) by native hCYP4B1 compared with rCYP4B1, despite their aforementioned high sequence identity [71]. This apparent absence of catalytic activity of the native hCYP4B1 has been attributed at least partially to the single amino acid alteration S427, while native rCYP4B1 contains the evolutionary highly conserved proline at the corresponding position 422 [24]. Replacement of serine with proline (h-S427P) restored hCYP4B1 activity toward 4-IPO **25**, but only partly. Therefore, S427 in hCYP4B1 cannot be solely responsible for its apparent inactivity. Following this observation, another twelve amino acids of rCYP4B1 that are critical for the high activity toward 4-IPO **25** and differ from the primary sequence of hCYP4B1 were identified, resulting in the aforementioned engineered h-P+12 variant that was as stable and active toward 4-IPO **25** as rCYP4B1 [40].

Another pneumo-protoxin associated with CYP4B1 is 3-methylindole (3-MI) **23**, which is a fermentation product of tryptophan from rumen and intestinal microorganisms and can also be found in cigarette smoke [73,74,75]. Lung-specific toxicity was observed mainly in cattle and goats but also to a lesser extent in rabbits and mice [75]. The toxic product of 3-MI **23** is a methylene imine, that is initially produced by several lung P450s, including CYP4B1, which catalyzes the desaturation of the methyl group of 3-MI **23** [76,77].

Two other pneumo-protoxins related to bioactivation by CYP4B1 are the cyclic arylamines 2-aminoanthracene (2-AA) **21** and 2-aminofluorene (2-AF) **22**. The P450-catalyzed bioactivation of these compounds leads to the formation of DNA-binding metabolites. Toxic metabolites can be formed, for example, via heteroatom oxidation or hydroxylation at the C1 carbon atom by CYP4B1 [78,79].

In order to find substrates for a CYP4B1-based suicide gene system, several potentially cytotoxic compounds were screened for conversion by rCYP4B1, as well as the reconstituted h-S427P and the engineered h-P+12 variants [62]. These exogenous compounds included the previously described CYP4B1 substrates 4-IPO **25**, 3-MI **23**, 2-AA **21**, and 2-AF **22**, as well as components with structural similarities to 4-IPO **25**, such as perilla ketone (PK) **26**, 2-furylpentyl ketone (2-FPK) **27**, 2-pentylfuran (2-PenF) **28**, 2-hexylfuran (2-Hex-F) **29**, and 2-heptylfuran (2-HepF) **30** (Figure 1b). In cytotoxicity assays in human cell lines, all tested components except 2-AF **22** and 2-HepF **30** were converted to a toxic species by at least rCYP4B1. PK **26** was found to be a tight binder to CYP4B1 (K_D_ = 0,25 µM) and was also the compound that was most extensively metabolized by oxidative processes to several non-reactive reaction products [62]. PK **26** is a natural furan that occurs in the essential oil of the mint plant *Perilla frutescens* [80] and is structurally very similar to 4-IPO **25**; the two compounds differ only in their hydrophobicity in that 4-IPO **25** has a terminal hydroxyl group while PK **26** has a methyl group on the corresponding position in its alkyl chain. Based on the knowledge that most P450s, and most likely CYP4B1, exhibit high hydrophobicity in the active site, it was hypothesized that the more hydrophobic substrate PK **26**, in contrast to 4-IPO **25**, can enter the active site in two different orientations, which was later supported by computational docking [23]. Nevertheless, PK **26** showed stronger toxicity than 4-IPO **25** in primary human T-cells using rCYP4B1 as well as h-P+12, indicating that the initially hydroxylated metabolites of PK **26** might also serve as CYP4B1 substrates and can be bioactivated on the furan ring in a second oxidative step [62]. In experiments with various mammals, the same pulmonary symptoms seen with 4-IPO **25** were also observed when PK **26** was administered [81,82,83].

In a further study, Kowalski et al. searched for synthetically accessible substrates that could be used analogously to 4-IPO **25** and PK **26** in CYP4B1-based suicide gene systems [29]. For this purpose, a series of *N*-alkyl-3-furancarboxamides **31**–**38** were synthesized in which the furan “war head” of 4-IPO **25** was retained, but the hydroxyl group at the end of the alkyl chain was removed and the alkyl chain lengths were varied between methyl (NC1-FCA) **31** to octyl (NC8-FCA) **38** (Figure 1b). Cytotoxicity assays demonstrated that there is a parabolic relationship between the alkyl chain length and toxicity, so that those derivates with moderate chain lengths (NC3-FCA to NC6-FCA) **33**–**36** showed similar cytotoxicities to 4-IPO **25**, while the toxicity of the shorter (NC1-FCA and NC2-FCA) **31,32** and longer (NC7-FCA and NC8-FCA) **37,38** derivatives was reduced (indicated by the red bar in Figure 1b). The authors suggested that this relationship may be due to the low affinity of CYP4B1 for short chain lengths and its regioselectivity for ω-hydroxylation, so that by using longer chain length substrates, CYB4B1 tends to hydroxylate the alkyl chain carbon atoms rather than to activate the furan ring (indicated by the blue bar in Figure 1b) [29].

## 4. Regulation of CYP4B1 Expression

In mice, 4-IPO **25** is a kidney and lung toxin in males but causes only lung damage in females. The gender-dependent toxicity in mice correlated with CYP4B1 expression, which is regulated by androgens in the kidney [84]. Due to gender differences observed in different species, androgen regulation at a transcriptional basis was analyzed. Thereby, Northern blot analysis revealed that after the castration of different mice strains, CYP4B1 transcription and thus mRNA levels decreased [85]. In addition, the castration of male rats resulted in a decreased CYP4B1 transcription level. This effect could partially be recovered by testosterone treatment, suggesting androgen-induced activation as well as sex-specific differences in CYP4B1 transcription in rats [86]. In contrast, no sex-specific differences in CYP4B1 mRNA expression in pig kidney or liver tissues were observed. Neither castration of male pigs nor testosterone propionate (TP) treatment significantly changed CYP4B1 mRNA levels among both sexes. However, TP treatment and castration had an impact on the mRNA levels of other P450s of family 4 (CYP4A24/25) [87]. These results suggest gender-specific differences in some species; however, there seem to be species differences as well. Possibly, these differences depend on host genetic factors and the concentration of serum androgen. In addition, it has been hypothesized that there is a tissue-selective factor responsible for the androgen-related expression of *p450*-genes [87].

In addition to androgen-dependent transcriptional regulation, hypoxia is involved in CYP4B1 expression. Southern blot analysis highlighted enhanced CYP4B1 cDNA signals in hypoxia-treated tissues compared to controls, thus suggesting an enhanced mRNA level of CYP4B1 under hypoxic conditions [57]. In addition, CYP4B1 was found to be involved in hypoxia-induced production of 12-HETE and 12-HETrE [57,88]. Furthermore, it is reported that clofibrate, an exogenous PPAR agonist, increased CYP4B1 cDNA levels and thus also mRNA in corneal tissue as well as 12-HETE and 12-HETrE synthesis, also suggesting an involvement of PPAR in CYP4B1 transcriptional regulation [57].

In addition to CYP4B1 transcriptional studies, numerous studies aimed at detecting transcription factor-binding sites and analysis of the promoter region have been performed. Supporting the androgen-related transcriptional regulation of CYP4B1, analyses of the promoter region also indicated an androgen-induced regulation. The testing of numerous CYP4B1 promoter constructs fused to the luciferase gene highlighted increased luciferase activity upon dihydrotestosterone treatment in mice. However, further observation of the promoter region revealed no androgen response element; thus, it was suggested that androgens only affected CYP4B1 regulation indirectly [85]. The potential role of CYP4B1 in 12-HETE and 12-HETrE production and the inflammatory response prompted examination of the CYP4B1 gene regulation in rabbit cornea [88]. Thereby, computational analyses of the 5′-flanking region of the rabbit corneal CYP4B1 promoter region revealed the presence of nuclear receptor motifs for retinoic X receptors (RXRs) as well as heterodimers formed by RAR/RXR, vitamin D receptor (VDR)/RXR, and peroxisome proliferator-activated receptor (PPAR)/RXR [88]. Furthermore, these analyses revealed VDR motifs in the 5′ promoter region, showing 80% sequence homology to the VDR element [88]. For promoter activity analysis, different luciferase reporter constructs were tested and luciferase activity was measured. Thereupon, the transcriptional activation of the CYP4B1 promoter was enhanced upon activation of RXR and RAR by 9-*cis*-retinoic acid and all-*trans*-retinoic acid treatment [89]. In contrast, treatment with vitamin D showed no significant effect; the authors suggested that vitamin D alone might be not sufficient to drive receptor dimerization and thus promoter activity [88]. Moreover, vitamin D is metabolized to its active metabolite, 1,25-dihydroxyvitamin D (calcitriol), that binds the VDR [90], which might drive receptor dimerization more efficiently. Corresponding to RXR/RAR activation, increased 12-HETE and 12-HETrE production under normal conditions was observed [88]. Thus, the effect of RAR activation on CYP4B1-mediated 12-HETE and 12-HETrE levels suggest a linkage between wound healing and inflammation at the ocular surface. Moreover, the CYP4B1 promoter region in rabbit cornea contains hypoxia-related transcription factors including hypoxia-inducible factor 1 (HIF-1), nuclear factor-kappa light chain enhancer of activated B cells (NF kB), and activator protein 1 (AP-1) [91]. The presence of a HIF-1 regulatory site clarifies the hypoxia-induced enhanced CYP4B1 transcription in mRNA studies. 

Furthermore, a comprehensive characterization of the hCYP4B1 promoter and the identification of potential binding sites for transcription factors have been performed [92]. This revealed several up- and downstream hCYP4B1 regulatory domains by transient expression of hCYP4B1/luciferase constructs in diverse cell lines including lung as well as liver carcinoma cell lines. Three main regulatory elements could be identified, a proximal positive, a distal, lung-selective positive, and a proximal, liver-selective negative regulatory element. Within the proximal positive acting regulatory element, two binding sites for Sephadex proteins (Sp)/Krüppel-like transcription factors, Sp1 and Sp3 were identified. Moreover, experiments in lung-derived cells highlighted the synergistic regulation of Sp1 transcription factors involving the upstream proximal and distal regulatory domains, and it was shown that Sp1 acts as a key regulatory element in the lung-specific expression of CYP4B1 by binding both the proximal and lung-selective distal enhancer region [92].

In summary, it appears that CYP4B1 expression can be altered, with most regulation occurring at the transcriptional level, and due to the tissue-selective enhancer and repressor elements, the lung-specific expression pattern of CYP4B1 could be demonstrated.

## 5. Tissue Distribution of CYP4B1, Single Nucleotide Polymorphisms, and Involvement in Several Cancers

CYP4B1 is an extrahepatic enzyme predominantly expressed in whole-lung tissues [31,93,94]. It has been detected in lung tissue from several species including rabbit, guinea pig, mouse, monkey, hamster, and rat. Enzyme expression correlated with the effects of anti-CYP4B1 on 2-AF **22** metabolism [95]. In addition to the high expression in lungs, the gene expression of CYP4B1 includes inter alia adipose tissues, as well as the bladder, breast, prostate, and uterus (https://gtexportal.org/home/gene/CYP4B1, accessed on 15 January 2023). However, the tissue distribution of CYP4B1 varies between different species. In rabbit bladder tissues, rCYP4B1 could be detected by immunoblotting as well as 2-AF **22** metabolization. Thereby, rCYP4B1 represented approximately 20% of the total P450s [95]. Furthermore, rCYP4B1 could also be detected in rabbit small intestine, cornea, colon, kidney, heart, brain, and gastrointestinal tissues [96]. In mouse, high levels of CYP4B1 were detected in the lung (especially in Clara cell-rich bronchi and bronchioles, while expression was absent from alveoli), brain (especially in the cortex), and kidney (with specific localization to the proximal convoluted tube), whereas CYP4B1 transcript levels were low in the spleen, testis, liver, and skeletal muscle [84,97]. In mouse ontogeny, CYP4B1 expression was analyzed during different developmental stages, including gastrulation (E7), neural patterning and somitogenesis (E11), organogenesis (E15), and the fetal period (E17). Agarose gels of CYP4B1 PCR-amplified products revealed that starting at E11, CYP4B1 is expressed in all stages of mouse ontology [97]. In humans, hCYP4B1 transcript levels were high in lung tissues, accounting for 70% of the total distribution. In general, it was shown that in human fetus and adults, hCYP4B1 levels were highest in the lungs; however, the amount of measured PCR product increased upon development. In the heart, skeletal muscle, and kidney, hCYP4B1 was only expressed poorly, and hCYP4B1 could not be detected in human fetal or adult liver [97]. Moreover, hCYP4B1 could also be detected in bladder microsomes, esophageal mucosa, and skin tissues [26,98,99].

For hCYP4B1, multiple single nucleotide polymorphisms (SNPs) are described. Online tools, such as https://www.ensembl.org, accessed on 15 January 2023), provide more than 6,000 SNPs for hCYP4B1. The data were collected based on three databases, namely, “The Human Gene Mutation Database” (HGMD; https://www.hgmd.cf.ac.uk/ac/index.php, accessed on 15 January 2023), “The Single Nucleotide Polymorphism Database (dbSNP; https://www.ncbi.nlm.nih.gov/snp/, accessed on 15 January 2023), and “The Catalogue of Somatic Mutations in Cancer” (COSMIC; https://cancer.sanger.ac.uk/cosmic, accessed on 15 January 2023). Most SNPs are located within introns, but more than 1000 SNPs are present within the coding sequence. However, only 14 SNPs have been mentioned to be of clinical significance, and further studies on hCYP4B1 SNPs are even more sparse. Analyses of the genetic polymorphism of French Caucasians and Japanese revealed the presence of six different allelic variants, CYP4B1*1–7 in addition to the native gene designated as hCYP4B1*1 [17,100,101]. The variants hCYP4B1*3–6 harbor missense mutations encoded on exon five, eight, or nine. The variants hCYP4B1*2 and hCYP4B1*7 are more interesting due to a deletion of two single nucleotides (A881-T882) leading to a frame shift and thus to a premature stop codon. Analyses of the genotype revealed that in addition to the native gene, the inactive hCYP4B1*2 allelic variant is pervasive, accounting for 33% of all alleles in Japanese and 13–15% in French Caucasian individuals. Moreover, 9% of the Japanese individuals and only 2% of French Caucasian individuals are homozygous for hCYP4B1*2 [100,101].

In a very recent study, three hCPY4B1 SNPs, namely, rs2297809, rs2297810, and rs4646491, were analyzed concerning their relationship towards gastric cancer in the Chinese Han population, which is one of the most common malignant tumors. It was shown that hCYP4B1-rs2297809 and hCYP4B1-rs2297810 had no effect on gastric cancer development, whereas hCYP4B1-rs4646491 was associated with an increased risk of developing gastric cancer [102].

High expression levels of CYP4B1 in the lungs and its known protoxin bioactivation capabilities generated hypotheses that the enzyme might be associated with lung cancer, and several studies on this topic have been published [103,104,105,106]. In addition, the genetic polymorphism of carcinogen-metabolizing enzymes is suggested to modify an individual’s susceptibility to lung cancer. Therefore, the correlation of the allelic variants hCYP4B1*1–7 between lung cancer cases and controls in the Japanese population was performed. However, no hCYP4B1 genotypes were associated with lung cancer risk; thus, genotype as well as allele frequencies showed no significant difference between the control group and cancer cases [107]. Strikingly, based on data from the Cancer Genome Atlas (TCGA) project and the Gene Expression Omnibus (GEO) database, analysis of hCYP4B1 expression in patients with lung adenocarcinoma (LUAD) showed that hCYP4B1 mRNA expression was significantly reduced in patients with LUAD [104,106]. Additionally, decreased hCYP4B1 expression was more frequently observed in patients younger than 65 years, those with a history of pharmaceutical or radiation therapy, showing mutations in signaling pathways including mutations KRAS, EGFR, or ALK, and with death [105]. In contrast, another study reported that hCYP4B1 mRNA is one of the top upregulated genes in LUAD samples [103]. Moreover, it was shown that hCYP4B1 mRNA is reduced 2.4-fold in lung tumor tissues compared to normal lung tissues. However, mRNA levels between several individual samples showed high variability, thus questioning biological relevance [25].

Some studies revealed the presence of CYP4B1 in bladder tissues, thus provoking studies on possible correlation with the risk of developing bladder cancer and hCYP4B1. It was found that CYP4B1 is expressed in rat bladder mucosa as well as in mouse bladder [108]. Experiments in rats highlighted sex- and age-dependent differences in the CYP4B1 expression pattern in the bladder. Thereby, CYP4B1 protein levels increased with development in male rats while in female rats, variations in CYP4B1 protein levels could not be detected with increased age, suggesting influences of androgens on CYP4B1 expression [86]. Moreover, the CYP4B1 protein was also detected in the human bladder by Western blot analysis. However, the expression of CYP4B1 in the human bladder exhibited high variation, whereby not only inter-individual but also differences between non-bladder tumor and bladder tumor tissues were noted. Thereupon, CYP4B1 expression in the non-bladder tumor tissue appeared to be lower compared to its expression in bladder tumor samples [26]. In contrast, it was found that downregulation of the CYP4B1 protein resulted in the development of urothelial carcinomas including urinary bladder and upper urinary tracts, leading to a poor prognosis [27]. Both the CYP4B1 expression level and its genotype impact the risk of bladder cancer. Genetic polymorphism analysis revealed that two allelic combinations, CYP4B1/*1/*2 and CYP4B1/*2/*2, are associated with an increased risk of bladder cancer in the Japanese population [109]; however, as mentioned above, CYP4B1*2 results in an inactive enzyme [101].

Other studies sporadically describe the influence of CYP4B1 in other cancer types including adrenocortical carcinoma, prostate cancer, and breast and ovarian cancer. In adrenocortical carcinoma, CYP4B1 expression was shown to be nearly absent, while CYP4B1 expression in adrenocortical adenomas was shown to be significantly reduced compared to normal adrenocortical samples. Moreover, gene expression analyses demonstrated the suppression of CYP4B1 in adrenocortical tumorigenesis [110]. CYP4B1 mRNA was also detected in human prostate samples [111,112]; 60% of the prostate cancer samples were positive for CYP4B1 mRNA while in non-cancerous prostate samples, only 50% were positive for CYP4B1 mRNA [112]. Similarly, CYP4B1 could also be detected in breast and tumor breast tissues, with 76.9% or 84.6%, respectively [113], thus suggesting an increased risk of developing prostate or breast cancer with increased CYP4B1 expression.

In summary, based on the knowledge collected so far, it appears that CYP4B1 is expressed in a variety of tissues, with the lungs being the predominant sites. The involvement of CYP4B1 in cancer has been implicated but has not been conclusively proven to date.

## 6. Development of a CYP4B1-Based Suicide Gene System for Cancer Treatment

In recent years, genetically engineered autologous T-cells have constituted powerful new immunological treatment strategies that provide hope for curative responses in patients with very poor prognosis of hematological malignancies of the B-cell lineage [114]. In an effort to cut down costs and to develop *off-the-shelf* products, a growing number of clinical trials with these adoptive T-cell therapies plan to use allogeneic T-cells as universal immune effector cells [115,116]. Allogeneic or haploidentical donor T-cells are also used after hematopoietic stem cell transplantation for relapsed/refractory or persisting leukemias [117]. For the clinical usages of not completely HLA-matched mature donor T-cells, safety systems that facilitate in vivo control of the genetically modified T-cells after infusion into patients are highly attractive [118,119,120]. One strategy to effectively control the side effects of adoptive T-cell therapy is to additionally equip the genetically engineered T-cells prior to infusion with a suicide gene that will enable directed and specific elimination of the donor T-cells in the patient [121,122].

The most studied suicide gene system in humans is the herpes simplex virus *thymidine kinase* gene (*HSV-tk*) whose product is capable of monophosphorylating the prodrug ganciclovir; monophosphorylated ganciclovir is then further processed by endogenous human enzymes into cytotoxic metabolites [117]. Although the *HSV-tk* suicide gene system has been successfully used in phase I/II clinical trials, some groups have reported that its utility is limited due to high immunogenicity of the viral protein leading to rapid elimination of HSV-tk-positive T-cells [123]. Therefore, other suicide gene systems have been developed and a few successfully tested in clinical settings [124,125].

As summarized above, CYP4B1 plays a major role in metabolizing exogenous protoxins and is the only enzyme in mammals that specifically catalyzes the metabolic activation of 4-IPO **25,** thereby generating highly reactive DNA alkylating products that cause acute cellular cytotoxicity [28,29,126]. As the mammalian CYP4B1 enzyme is predominantly expressed in Clara cells and type II pneumocytes of the lung, the injection of 4-IPO **25** into laboratory animals, such as female mice, rabbits or dogs, led to rapid activation of the protoxin in these lung cells and resulted in lethal acute respiratory distress of the animals within hours [66,127,128]. Based on two publications from the Developmental Therapeutics Program of the National Cancer Institute (NCI) of the U.S.A. in 1986 and 1990 [129,130], partly reporting divergent data on the same cell lines, human lung cancer cell lines and primary human lung cancer cells were reported to variably possess the metabolic enzyme fittings necessary for the bioactivation of 4-IPO **25** to cytotoxic metabolites. Therefore, 4-IPO **25** was strongly promoted by the NCI Developmental Therapeutic Program as a new ‘targeted’ chemotherapeutic drug that could predominantly induce cytotoxicity in human lung tissue at doses not affecting liver or kidney cells [127]. However, already by 1991, it was demonstrated by the Gonzalez laboratory at the NCI that hCYP4B1 simply cannot metabolically activate 4-IPO **25** [71]. This finding is in line with all functional data ever published for CYP4B1 [31,62,131]. Retrospectively, it therefore is quite difficult to understand why three clinical phase I/II trials with 4-IPO **25**, published in 1993, 1998, and 2001, were still conducted in patients with lung or hepatic cancers [132,133,134], as hCYP4B1 cannot activate 4-IPO **25**, and other P450 enzymes in humans seem to never have adapted that function [31,71]. In hindsight, it is not a surprise that no anti-tumor effect in lung was obtained, but instead liver toxicity was observed in the clinical studies [132,133,134].

**Figure 3:**
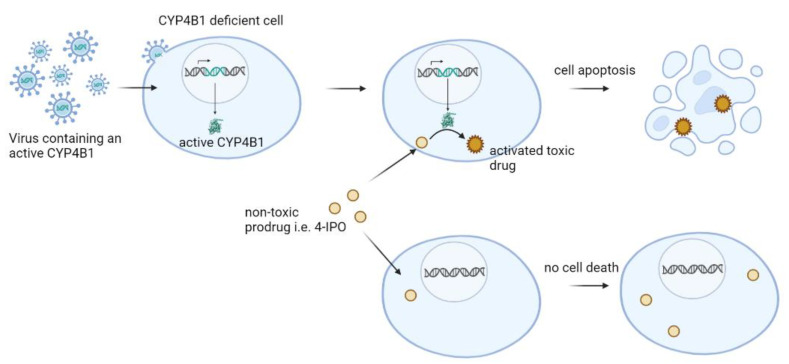
Schematic representation of a CYP4B1-based suicide gene system. CYP4B1-deficient cells are transduced with integrating viral vectors to express an active CYP4B1 protein. This enzyme converts a non-toxic substance (protoxin) into a cellular toxin only in CYP4B1-positive cells.

Based on the fact that the endogenous hCYP4B1 is not active against 4-IPO **25**, Rainov et al. first suggested the use of the highly active rCYP4B1 in combination with 4-IPO **25** as a suicide gene system (Figure 3) to treat brain tumors [135]. In vitro experiments showed that only low concentrations of 4-IPO **25** were required to kill human and rat tumor cells that expressed rCYP4B1 with a direct correlation between the rCYP4B1 protein concentration and toxicity. Surprisingly, the growth of rCYP4B1-expressing tumor cells that were implanted subcutaneously in mice was stopped by intraperitoneal administration of 8 mg/kg 4-IPO **25** daily for nine consecutive days, while no major lung or liver toxicity was observed [135]. Further, removal of the 4-IPO **25** after a continuous 24 h treatment had no permanent cytotoxic effect on the surviving transgene-negative cells [28]. Comparing the rCYP4B1/4-IPO system to the HSV-tk/Ganciclovir system in vitro, a significantly faster induction of apoptosis independent from the cell cycle status of the suicide gene expressing cancer cells was noted for the first combination [28,136]. The rCYP4B1/4-IPO system also did not exhibit any bystander activity, thus making it an interesting candidate suicide gene system [28,136]. However, the use of the rCYP4B1 isoform in humans in vivo may cause immunogenicity and rejection of the genetically engineered cells, similarly to what has been described for the HSV-tk/Ganciclovir system in humans [137].

To address any potential immunogenicity when planning to use an active CYP4B1 protein in patients, our research group initiated systematic amino acid substitution studies to activate hCYP4B1 with only minimal sequence changes [40]. The h-S427P exchange improved the protein stability, but only partially restored the activity of hCYP4B1, and a further twelve amino acid sequence alterations (h-P+12) were necessary to increase the activity level of the human enzyme to that of rCYP4B1 [40]. Importantly, all twelve amino acid substitutions are present in other human CYP4 family members at corresponding positions (Figure S3) [40], thus probably minimizing the immunogenicity of the h-P+12 protein.

rCYP4B1 as a prototypic active mammalian protein shows a rather narrow substrate spectrum; it is, however, known to specifically convert other nontoxic substrates such as PK **26 [83]** or 2-AA **21** [28] into cellular toxins [31]. 2-AA **21** can serve as a relatively specific substrate for CYP4B1 enzymes, introducing cell death in different glioma cell lines, including rodent 9L, human U87, or rat C6 cells [28,126,135]. However, 2-AA **21** belongs to a class of aromatic amines that are known mutagens and carcinogens [28,31], therefore rendering 2-AA **21** unsuitable as a protoxin for clinical application. As PK **26** induced severe pulmonary toxicity in laboratory animals and livestock [66,81,82,138], our research group systematically compared its cytotoxicity to 4-IPO **25** in rCYP4B1- and h-P+12-expressing HepG2 cells in vitro [62]. Cell death achieved with PK **26** and 4-IPO **25** was similar, but PK **26** efficiently induced cell death at a much lower concentration than 4-IPO **25**, with no PK **26** toxicity evident in control HepG2 or primary human T-cell cells [22,62]. Similar to 4-IPO **25**, PK **26** does not induce bystander effects when using HepG2 cells transduced with different CYP4B1 isoforms [62], whereas, for example, 2-AA **21** leads to a strong bystander activity [28,135]. We also tested other furan analogs, namely, 2-FPK **27**, 2-PenF **28**, 2-HexF **29**, and 2-HepF **30** (Figure 2b), in HepG2 cells that expressed rCYP4B1, h-S427, h-S427P, as well as h-P+12 [62]. Here, only the first two substrates were specifically processed by the active CYP4B1 enzymes to cause cellular toxicity, albeit to a much lower degree than 4-IPO **25** or PK **26** [62].

In summary, 4-IPO **25** and PK **26** seem to be excellent protoxin candidates for human applications; however, their high degree of hydrophobicity might require specific formulation strategies to be able to function in combination with h-P+12 as a suicide gene system in adoptive cellular therapies in patients.

## 7. Conclusions and Future Directions

CYP4B1 isoforms from different mammals vary in their amino acid sequence and consequently also in their activity in processing substrates, such as 4-IPO **25**. hCYP4B1 is a clear outlier among the mammalian CYP4B1 isoenzymes because it contains the proline-to-serine alteration p.P427S. From an evolutionary genetics point of view, it is known that hCYP4B1 acquired the p.P427S alteration at the transition of great apes to humanoids. This alteration seems, however, not be the sole reason for the loss of activity, as it has been shown that the single amino acid change reversing the alteration in hCYP4B1 (p.S427P) only partially restores its capability to convert 4-IPO **25** and unsaturated fatty acids. In addition, the proline-to-serine alteration does reduce—but not completely abolish—metabolic activity when introduced into highly active CYP4B1 isoforms, such as rCYP4B1 (p.P422S). Sequence variations in other regions seem to be important for activity as well; 50 years after the discovery of CYP4B1, however, its evolution is still largely unstudied, and the primary biological functions of CYP4B1 proteins remain unclear. Consequently, it is important to fully understand the primary biological functions of CYP4B1 and to identify and evaluate CYP4B1 sequence variations against an evolutionary background to pinpoint at which stage(s) during evolution changes occurred that led to change/loss/acquisition of activities. In addition, the reconstruction and characterization of the CYP4B1 ancestral sequence might shed light on its historical function and evolution, as, for example, recently demonstrated for CYP1B1 [139] or CYP11A1 [140].

Although there are reports on the roles of CYP4B1 in the initiation and progression of cancer, the picture is far away from being complete, and further investigations are needed. Despite this, the development of a suicide gene system based on hCYP4B1 is a promising strategy to develop targeted therapies for cancer treatment. In the future, more compounds should be tested to identify alternative agents for this approach.

## Data Availability

Not applicable.

## Supplementary Materials

The following supporting information can be downloaded at https://www.mdpi.com/article/10.3390/ijms24032038/s1, Figure S1: Close-up of the residues that line the binding pocket in hCYP4B1; also shown is the energetically most favorable binding position of 4-IPO **25** obtained from molecular docking; Figure S2: Multiple sequence alignment of mammalian CYP4B1 enzymes obtained with the MAFFT algorithm of JalView. All sequences were obtained from the UniProt database (https://www.uniprot.org/, sequences of the same enzymes that have been deposited in various databases can differ). Secondary structural elements were adapted from the corresponding positions in the crystal structure of rCYP4B1 (5T6Q.pdb); Figure S3: Multiple sequence alignment of the human CYP4 family obtained with the MAFFT algorithm of JalView. Secondary structural elements were adapted from the corresponding positions in the crystal structure of rCYP4B1 (5T6Q.pdb).
