# Supplementary figures and images for: Spotlight on CYP4B1
Source: Int J Mol Sci. 2023 Jan 20;24(3):2038. doi: 10.3390/ijms24032038 (PMC9916508; doi:10.3390/ijms24032038)

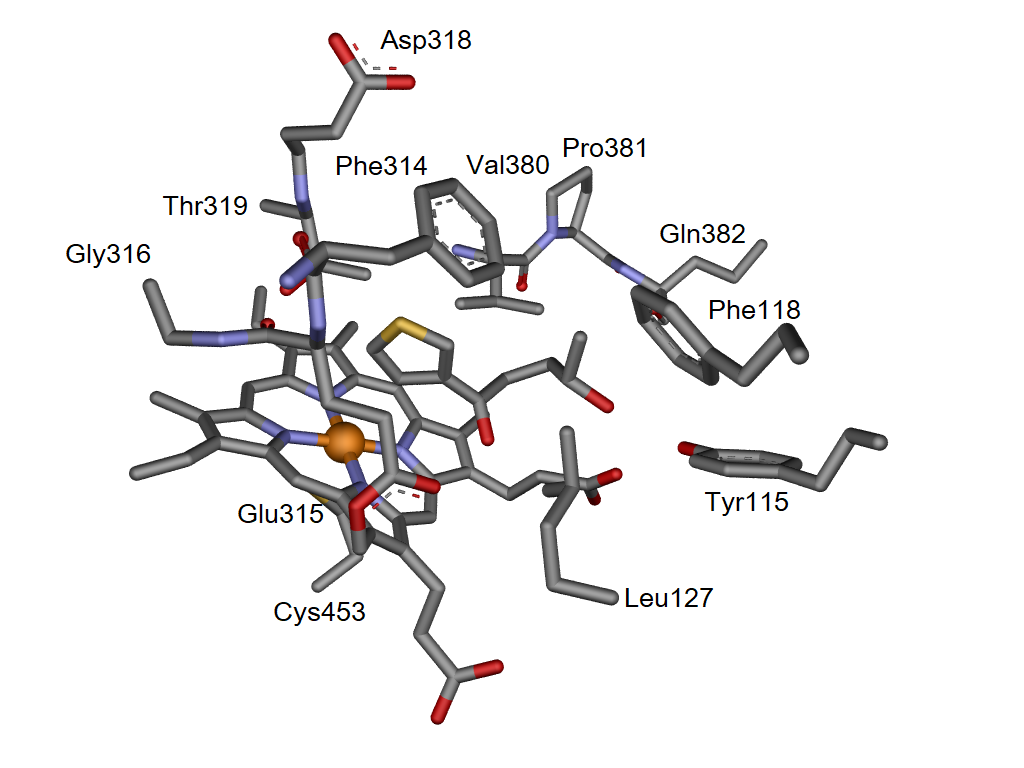

Supplement: Supplementary file 1 [file ijms-24-02038-s001.zip › Supplementary Figure_S1.png]

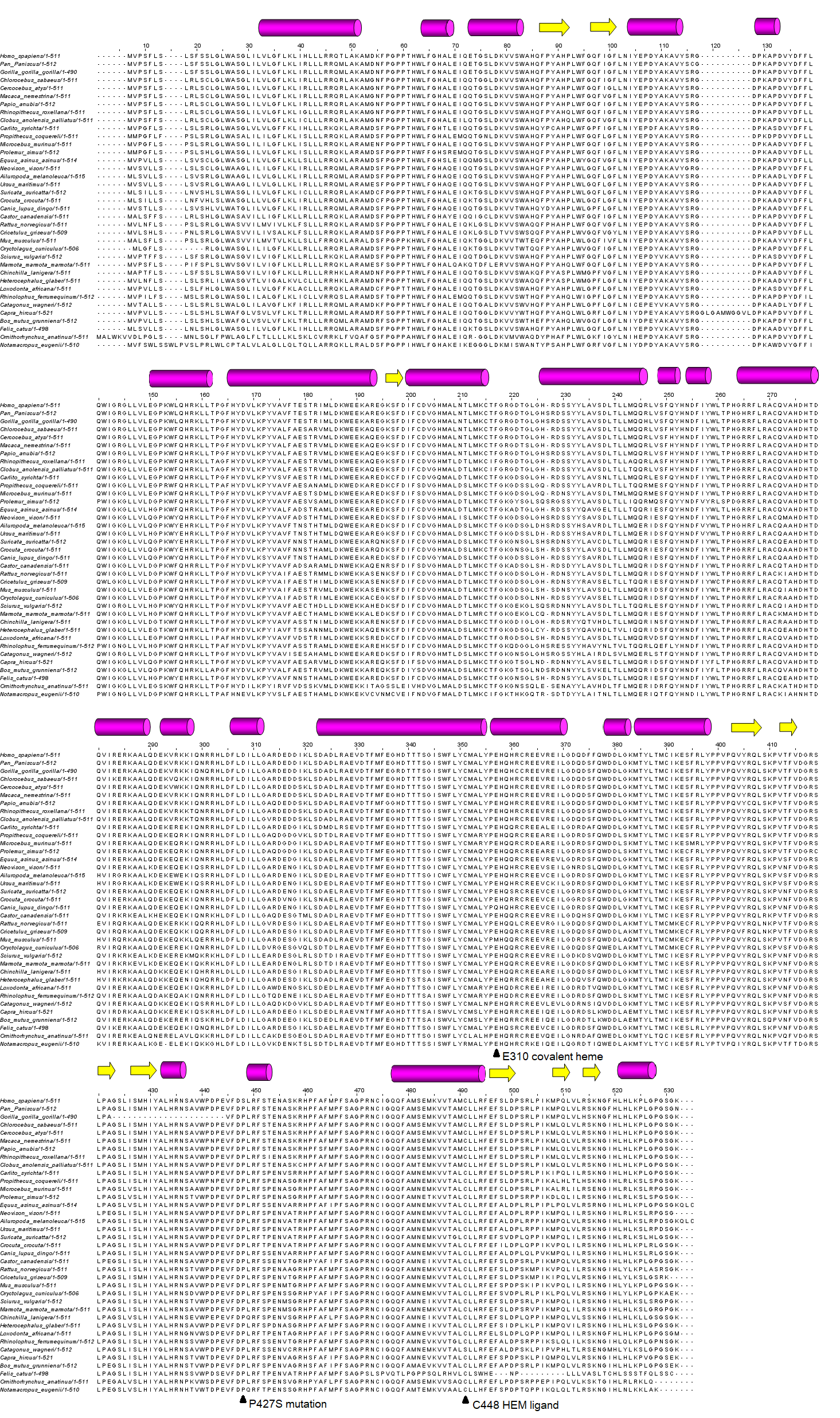

Supplement: Supplementary file 1 [file ijms-24-02038-s001.zip › Supplementary Figure_S2.png]

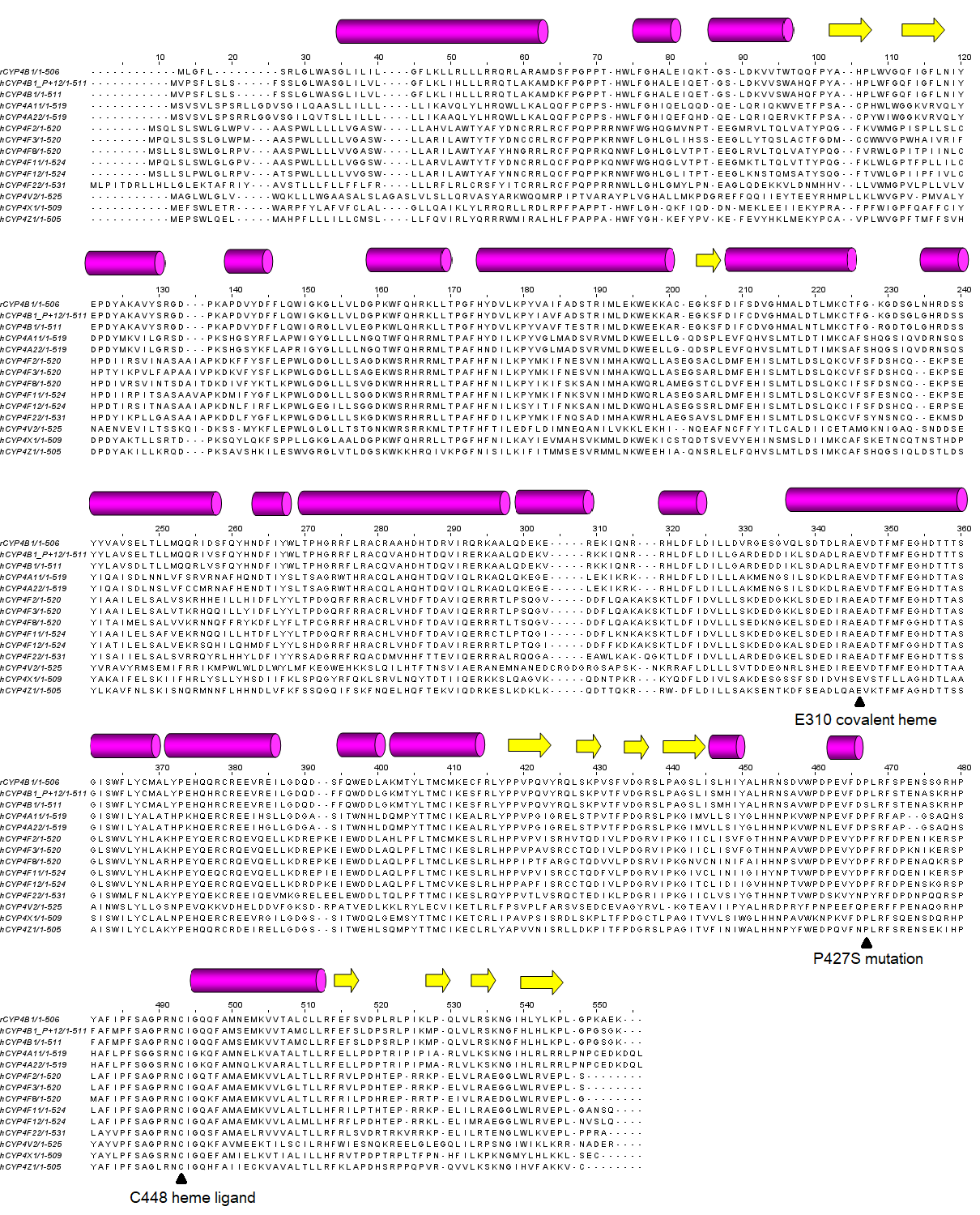

Supplement: Supplementary file 1 [file ijms-24-02038-s001.zip › Supplementary Figure_S3.png]
